# Genetic characterization of zoonotic *Dirofilaria asiatica* in Cambodian dogs through nanopore metabarcoding

**DOI:** 10.1128/spectrum.01990-25

**Published:** 2025-09-17

**Authors:** Lucas G. Huggins, Ushani Atapattu, Virak Khieu, Rebecca Traub, Vito Colella

**Affiliations:** 1 Melbourne Veterinary School, Faculty of Science, University of Melbourne, Parkville, Australia; 2 National Centre for Parasitology, Entomology and Malaria Control, Ministry of Health, Phnom Penh, Cambodia; University of São Paulo, São Paulo, Brazil

**Keywords:** next-generation sequencing, metabarcoding, One Health, Southeast Asia, filarioids, vector-borne pathogens

## Abstract

Filarioid nematodes are harmful vector-borne pathogens that afflict humans and other animals, with numerous species transmissible from animals to humans, i.e., zoonotic. In the last decade, one zoonotic filarioid, previously named *Dirofilaria* sp. Hong Kong genotype has been increasingly found as a cause of disease in people traveling from South Asia and has recently been characterized as a novel species: *Dirofilaria asiatica*. Nonetheless, the epidemiology of this parasite in Asia remains poorly understood. Therefore, with the aid of a novel pan-filarioid nanopore-based metabarcoding assay, we aimed to elucidate the epidemiology of dirofilariosis in Cambodia. We tested 504 canine blood samples collected from five regions and used the cytochrome c oxidase (*cox1*) gene sequences obtained to conduct phylogenetic analyzes. We found no evidence of the canine heartworm *Dirofilaria immitis* nor *Dirofilaria repens* in the country. Conversely, we detected *D. asiatica* and genetically characterized it from the eastern district of Tbong Khmum at a local prevalence of 4% (95% CI = 1.6%–9.8%), an area just ~100 km northeast of the country’s capital, Phnom Penh. We also detected *Acanthocheilonema reconditum* from 0.2% of dogs sampled (95% CI = 0%–1%). Phylogenetic and haplotype network analyzes of Cambodian *D. asiatica* sequences clustered with strong support to those found from canines and humans in Bhutan, Hong Kong, India, and Sri Lanka. This genetic characterization of *D. asiatica* in Cambodia highlights the need for further epidemiological research into human filariases in Cambodia, given the zoonotic nature of this parasite and that mass drug administration targeting filarioids is no longer conducted in the country.

**IMPORTANCE:**

Parasitic filarioid nematodes are transmitted by blood-sucking arthropods and can cause significant disease in animals and humans (i.e., zoonotic). One such zoonotic filarioid (*Dirofilaria asiatica*) that has recently been characterized has been found responsible for a growing number of cases of human filariases, particularly in individuals who live in or have traveled to South and Southeast Asia. *D. asiatica* predominantly infects dogs, and therefore, to better understand this parasite’s distribution, we tested 504 dogs from five regions of Cambodia using an advanced diagnostic approach. We found that the local prevalence of *D. asiatica* in dogs in the eastern district of Tbong Khmum was 4%. Given that mass drug administration programs to control human-infecting filarioid nematodes in Cambodia have stopped, this first detection of *D. asiatica* in Cambodia is alarming, given its potential to cause disease in vulnerable cohabiting people.

## INTRODUCTION

A diverse range of filarioid nematode species infect the domestic dog (*Canis lupus familiaris*), generating a spectrum of pathologies ranging from benign infections through to potentially fatal disease (1–3). For example, *Acanthocheilonema reconditum* generates a typically non-pathogenic infection in dogs, whilst canine heartworm (*Dirofilaria immitis*) can be lethal (1, 4). Crucially, many of these canine-infecting filarioids are also zoonotic and can infect humans as accidental hosts, leading to pronounced disease (5–10). Different culicid mosquito species within the genera *Aedes*, *Anopheles*, and *Culex* are some of the most important vectors of canine filarioids, whilst their propensity to take blood meals from other mammalian hosts, such as humans, provides an easy route by which these pathogens can be zoonotically transmitted (3, 11–13). Filarioid nematodes of the genus *Dirofilaria* are the species most frequently found causing zoonotic filariases, with three canine-infecting species, *D. immitis*, *Dirofilaria repens,* and the recently described *Dirofilaria asiatica* being the most common taxa responsible (8, 13–17). In addition, some rarer raccoon and bear-infecting species, amongst others, do occasionally generate clinically relevant infections in people (13, 18–20).

A growing number of studies have identified the importance of a novel *Dirofilaria* as a significant cause of zoonotic filariasis in Asia (3, 5–8, 16, 21). Since this species’ initial identification as *Candidatus Dirofilaria* hongkongensis in 2012 (14), it has been detected with increasing frequency in countries such as India (8), Sri Lanka (3), and Thailand (21), whilst additional cases outside of tropical regions have been found in travelers who have visited these nations (6, 7). Such increases in the detection of this filarioid may be due to various factors, including the use of improved molecular diagnostic tools together with a greater recognition of this species’ existence (16). A recent detailed morphological description of males, females, and microfilariae of this parasite, together with the characterization of its mitochondrial genome, enabled this species to be newly described as *D. asiatica* (16). Importantly, *D. asiatica* shares many morphological similarities to *D. repens*; however, it is genetically divergent, i.e., a cryptic species, and appears to have its own distinct distribution (16). The recent elucidation of the role dogs play as a reservoir host for *D. asiatica* is also significant, given that these animals do not experience overt disease, allowing the maintenance of this pathogen within a given population (3).

Cambodia is a Southeast Asian nation with a large population (~5 million) of free-roaming and semi-domesticated dogs, many of which are unowned and lack access to veterinary care (22–24). Previous studies have identified numerous vector-borne pathogens (VBPs) as being hyperendemic and diverse within local dogs (22); however, prior exploration of filarioids within Cambodian canine populations has been extremely limited (22, 25). Therefore, this study set out to comprehensively characterize the diversity and prevalence of canine dirofilariosis from five different and previously unexplored Cambodian provinces using an advanced nanopore-based metabarcoding sequencing method (26).

## MATERIALS AND METHODS

### Study sites, sample size calculations, and sample collection

**Fig 1:**
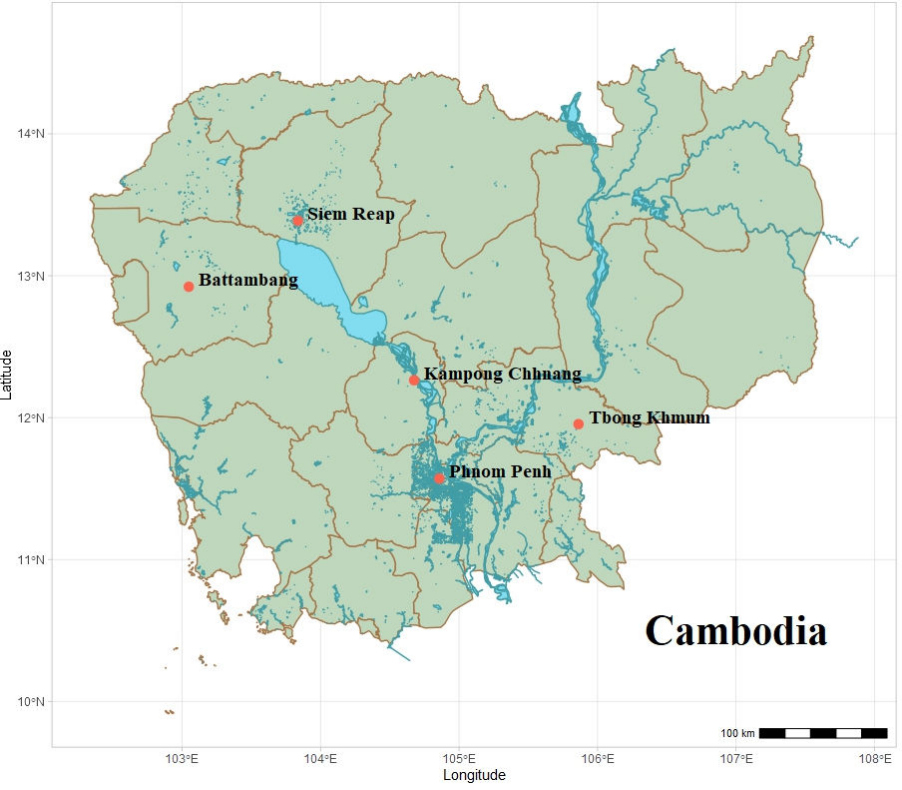
Map of field sites investigated in Cambodia. Turquoise shows waterways, light blue shows lakes, and brown lines delineate provinces. Samples were collected from Phnom Penh (PP; *n* = 149), Siem Reap (SR; *n* = 125), Kampong Chhnang town (KC; *n* = 48), Battambang city (BB; *n* = 100), and Tbong Khmum district (TB; *n* = 100). Map created in RStudio Version 2024.04.1.

A cross-sectional study was conducted across four provinces and one municipality in Cambodia as previously described by Huggins (22). A required sample size of at least 207 dogs was calculated using the formula *n* = z^2^p(1−*P*)/d^2^ where n is the required sample size, z (1.96) is the standard deviation at 95% CI, *P* is the expected prevalence (16%) based on Inpankaew (25), and d is the allowed relative error corresponding to effect size (0.05) (27). Samples were collected across April to May and September 2019, thereby encompassing the country’s drier, warmer northeast monsoon season and the wetter southwest monsoon period. A total of 504 canine blood samples were collected, including *n* = 149 from metropolitan Phnom Penh (11°31’N, 104°55’E) Cambodia’s capital city, *n* = 48 from Kampong Chhnang town (12°15’N, 104°39’E), *n* = 107 from Siem Reap city (13°26’N, 103°45’E), *n* = 100 from Battambang city (13°25’N, 103°73’E), and *n* = 100 from Tbong Khmum district (12°01’N, 105°30’E) see Fig. 1. Dogs were humanely restrained, and samples were only taken following informed consent from the owner. Relevant canine metadata, including age (as assessed by dentition), sex, breed, whether neutered, reproductive status, and sampling location coordinates were recorded by a veterinarian. The presence of ectoparasites, such as fleas, ticks, and lice, was also noted. A total 2 mL of whole blood was collected by a veterinarian via cephalic or jugular puncture into an anti-coagulation ethylenediaminetetraacetic acid (EDTA) tubes and temporarily kept on ice. After field work, samples were frozen at −20°C and shipped to the University of Melbourne for further analyzes.

Fieldwork in Cambodia was conducted under the University of Melbourne Animal Ethics Committee-approved Permit: 1814620.1.

### DNA extraction

Canine blood samples were defrosted, and total DNA was extracted from a 200 µL aliquot using the DNeasy Blood & Tissue Kit (Qiagen, Hilden, Germany) following the manufacturer’s protocol. The only modifications to this protocol were a 30 min proteinase K digestion at 56°C and two final elution steps, the first in 30 µL and the second in 20 µL (50 µL total eluent). Extracted DNA was kept at −20°C until further use.

### Filarioid worm pre-screening and metabarcoding analysis

To prevent deep-sequencing of filarioid-negative samples, pre-screening with a conventional polymerase chain reaction (cPCR) assay was used to assess whether a given sample was positive for any filarioid (see below). If a sample showed a band on a gel, then it was taken forward for filarioid metabarcoding. All 504 blood extracted DNA samples were pre-screened using 25 µL PCRs consisting of 12.5 µL OneTaq^®^ 2× Master Mix (New England Biolabs, Ipswich, USA), 1 µL of both the COIfilF and COIfilR primers (28), and 2 µL of template DNA. Thermocycling conditions were modified to: 1 cycle of 95°C for 40 s, 40 cycles of 95°C for 40 s, 50°C for 45 s, and 68°C for 45 s, with a final extension of 68°C for 5 min. Expected product size was ~650 bp. Two positive and one negative control were run for all pre-screening PCRs in batches of ~48 samples. Positive controls for pre-screening used *D. immitis* extracted DNA. Gel electrophoresis using 1.5% agarose gels was conducted on PCR products at 90 V for 45 min, with bands visualized on a ChemiDoc Imaging System (Bio-Rad, California, USA). If a band was visualized (including multiple bands and bright smears within the expected amplicon size range), then the samples were sent forward for metabarcoding. However, amplicons generated by this pre-screening PCR were not used for metabarcoding library preparation. Instead, samples found positive through pre-screening were processed for metabarcoding from the beginning of the protocol as per (26).

In total, 96 pre-screened blood extracted DNA samples, including one DNA extraction negative control, three PCR negative controls, and three positive controls (26), were processed for metabarcoding on Oxford Nanopore Technologies’ MinION Mk1b device. Sequencing was conducted on a Legion 7i Gen six laptop (Lenovo, Quarry Bay, Hong Kong) for between 48 h, i.e., until a sufficient sequencing depth had been achieved for all multiplexed samples (26). FAST5 files were then base-called using the super high-accuracy base-calling model with barcode removal using Guppy version 6.4.6.

All nanopore sequencing data produced in this study are available from the NCBI BioProject database BioProjectID PRJNA1102149; specifically, Sequence Read Archive (SRA) accessions SRX24327133 to SRX24327228.

### Bioinformatics

All bioinformatic processing was conducted using the NanoCLUST (29) pipeline using the parameters and reference database detailed in (26). Read thresholds to determine whether a sample was positive for a filarioid pathogen were calculated as per (22). In brief, uniquely identifiable positive control reads found in samples other than the positive control defined the read cut-off threshold for determining pathogen positivity or negativity.

### Statistical analyses

The 95% confidence intervals (CIs) for the frequency of detection of given filarioid pathogens were calculated using the Wilson score interval via the open-source software Epitools (https://epitools.ausvet.com.au).

### Phylogenetic analysis

Relevant published and verified filarioid mitochondrial *cox1* genes were downloaded from GenBank, imported into Geneious Prime 2023.2.1, and aligned with the relevant sequences obtained from our metabarcoding method, using MAFFT. Alignments were exported as FASTA files, which were subsequently converted to the NEXUS format with MEGA 11 (version 11.0.13) (30). The best nucleotide substitution model for each alignment was determined with maximum likelihood analysis, including 1st, 2nd, and 3rd codon positions in MEGA 11. The model with the lowest Bayesian information criterion value was selected for phylogenetic inference.

For Bayesian phylogenetic inference (BI), selected FASTA alignments were converted to NEXUS format for MrBayes (31) that includes a code block with instructions for Bayesian inference. This inference was performed using MrBayes 3.2.7 (32) on the alignment. Each BI was performed with 2 million Markov Chain Monte Carlo (MCMC) generations, sampling every 100th generation with four chains by allowing for transitions and transversions with gamma-distributed rates. Phylogenetic inference by the Neighbor-Joining (NJ) distance method was performed in MEGA 11 (version 11.0.13). The NJ analysis was performed with 2,000 bootstrap replications using the relevant model, based on best nucleotide substitution models as determined above, including both transition and transversions with gamma-distributed rates. Trees outputted by MrBayes were imported into FigTree (version 1.4.4.) and then Adobe Illustrator (version 27.3.1) for editing to improve clarity.

### Sequence type networks

A sequence-type network was constructed by considering each unique *Dirofilaria* mitochondrial *cox1* sequence based on species and location from the GenBank database as of the 30 August 2024. The selected sequences were aligned in Geneious Prime 2023.2.1 as described above and exported as a NEXUS file. Location information for each sequence was manually added to the NEXUS file in a ‘trait block’. A Minimum Spanning Network (MSN) (at epsilon = 0) was then constructed using PopART version 1.7 (33). An additional sequence-type network was created using only *D. asiatica cox1* sequences, following the same methodology. The design of the resulting networks was enhanced for clarity using Affinity Designer 2 Version 2.2.1.

## RESULTS

### Sample collection

A total of 504 dogs were sampled from five different field sites across Cambodia. The demographic composition of canines sampled was 229 (45.4%) females, 264 males (52.4%), and 11 (2.2%) unreported (Table 1). Most dogs were either puppies below 6 months of age, 154, (30.6%) or adults between 1 and 7 years old, 221 (43.8%). Nearly all dogs across all study sites were mongrels 492 (97.6%), with just 12 (2.4%) pure breeds, which included an Alsatian, a Golden Retriever, an English Bulldog, a Labrador, Huskies, and German Shepherds. The presence of ectoparasites, such as ticks and fleas, was more common than their absence, with 374 (74.2%) dogs found infested, compared to 130 (25.8%) dogs found without them.

**TABLE 1:** Study site (PP, Phnom Penh; KC, Kampong Chhnang; SR, Siem Reap; BB, Battambang; TK, Tbong Khmum), canine demographic data (F, female; M, male), and presence of ectoparasites (ticks and fleas) across the 504 canine samples assessed for filarioid pathogens by our metabarcoding assay^*a*^

	Sex			Age (years)					Breed		Ectoparasites	
Study Area	F	M	NA	< 0.5	> 0.5 to <1	> 1 to <7	> 7	NA	Mongrel	Pure	Present	Absent
PP (*n* = 149)	60	85	4	76	23	40	5	5	145	4	106	43
KC (*n* = 48)	25	23	0	12	5	31	0	0	46	2	37	11
SR (*n* = 107)	41	63	3	22	16	64	4	1	106	1	72	35
BB (*n* = 100)	49	48	3	23	16	54	6	1	95	5	72	28
TK (*n* = 100)	54	45	1	21	37	32	4	6	100	0	87	13
Total (*n* = 504)	229	264	11	154	97	221	19	13	492	12	374	130

^*a*^ For some dogs, sample data were not collected or not available (NA).

### Filarioid nematode pre-screening

A total of 89 of the 504 pre-screened samples showed bands when visualized on a gel. Not all bands were at the expected amplicon size for the cPCR used; nonetheless, in the interests of ensuring that all potential filarioid positive samples were deep-sequenced, these were sent forward to be analyzed by the metabarcoding assay. These were sequenced alongside seven control samples (three positive and four negative).

### Bioinformatic processing

Sequencing was stopped on the MinION device after 48 h at which point the number of active nanopores was <20 and therefore sequencing output was negligible.

A total of 3,193,891 raw reads (87.01 GB total data, i.e., FAST5 and FASTQ) were obtained. Most data passed initial quality control filtering, i.e., met a Q-score of ≥8, with a ratio of passed to failed bases of 3.68 Gb:1.38 Gb. The raw reads obtained were processed by the NanoCLUST bioinformatic pipeline into 2,771,437 filtered and polished reads, i.e., 86.7% of the total raw reads were used by the pipeline for further analysis. Most reads obtained were from positive control samples (95.7%), with the three positive controls attaining a total cumulative read count of 2,763,397 (2,652,886 post-filtering with NanoCLUST). For biological samples, raw read counts were much lower, ranging from 86 to 35,560 with a mean sample read count and standard error (S.E.) of 4,798±727. After filtering, biological sample read counts ranged from 0 to 27,802 with a mean and S.E. of 1,329±444.

### Filarioid nematode metabarcoding

Across the complete metabarcoding sequencing data set obtained only sequences characterized by NanoCLUST with an identity score of over 95% to a filarioid were extracted from the overarching data set and compiled alongside the relevant read counts (34). Sequences with an identity score of less than 95% to a known filarioid were investigated further via blastn analysis to the full GenBank database. If they were found to match sequences from an off-target organism, then they remained excluded. These off-target sequences typically belonged to DNA amplified from the canine host’s genome. The filarioid pathogens identified were *A. reconditum* (one dog) and *D. asiatica* (four dogs), see Table 2. Therefore, across all field sites, 0.2% (95% CI = 0%–1%) of dogs were found to be infected with *A. reconditum,* and 0.8% (95% CI = 0.3%–2%) were infected with *D. asiatica*. Overall, 1% of dogs (95% CI = 0.4%–2.3%) were infected with a filarioid. No canines were found to be coinfected with both pathogens. Filarioid infections were not evenly distributed between study sites, with all *D. asiatica* infections identified in the district of Tbong Khmum at a prevalence of 4% (95% CI = 1.6%–9.8%).

**TABLE 2:** Results for the five canines found positive for a filarioid pathogen using the filarioid *cox1* gene targeting metabarcoding assay^*a*^

Sample code	BB018	TK004	TK019	TK031	TK043
Filarioid detected	*A. reconditum*	*D. asiatica*	*D. asiatica*	*D. asiatica*	*D. asiatica*
Top NCBI accession number hit	JF461456.1	NC_031365.1	PP158772.1	PP158772.1	PP158772.1
Identity (%)	99.69	99.56	99.7	99.7	99.85
Query cover (%)	97	100	100	99	100
Length (bp)	664	674	667	667	668
Total post-filter reads per sample	3,915	27,802	15,580	5,672	22,463
Filarioid reads in sample (%)	2,509 (64.1%)	27,732 (99.7%)	15,071 (96.7%)	4,857 (85.6%)	22,283 (99.1%)
Study site	Battambang	Tbong Khmum	Tbong Khmum	Tbong Khmum	Tbong Khmum
Host sex	Male	Male	Male	Male	Female
Host age (years)	>7	>0.5 to <1	>1 to <7	>0.5 to <1	>7
Host breed	Mongrel	Mongrel	Mongrel	Mongrel	Mongrel

^*a*^ Identity and query cover scores, as well as NCBI accession numbers, were obtained by classifying metabarcoding consensus sequences against NCBI’s GenBank using blastn. Total post-filter reads per sample refers to the total number of sequences retained after filtering with NanoCLUST.

### Phylogenetic analysis

**Fig 2:**
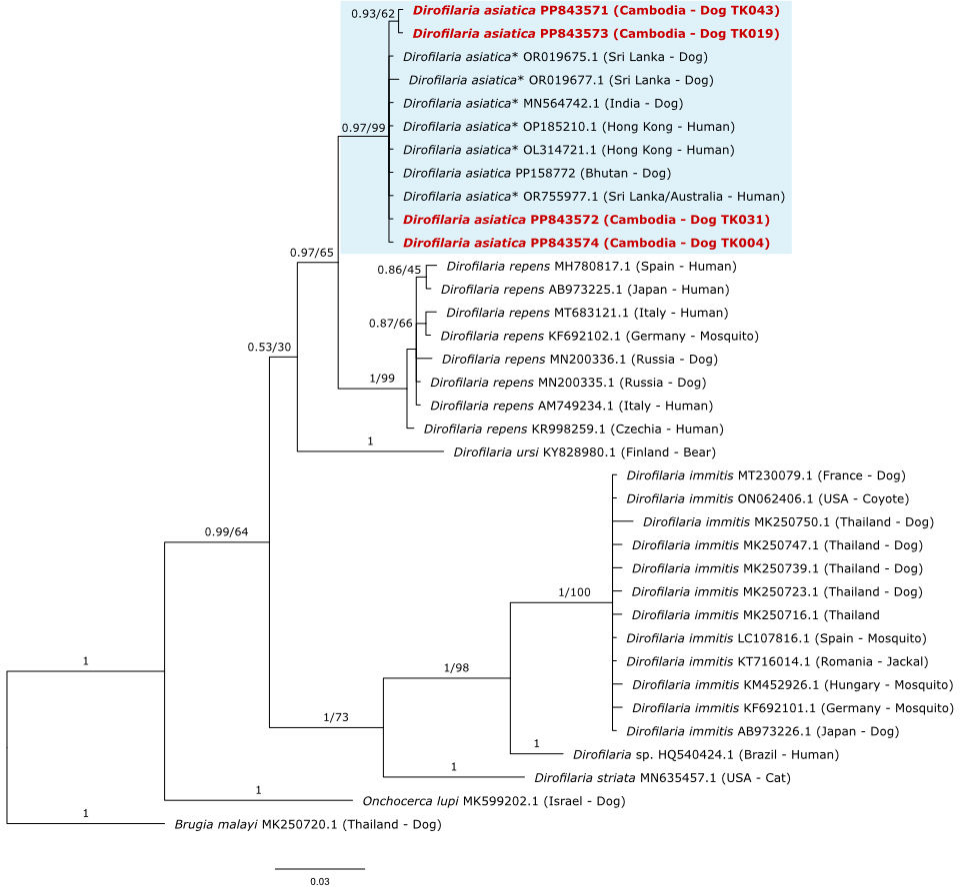
Phylogenetic relationship of the four *D. asiatica* (red and bold) detected from the blood of Cambodian dogs, alongside representative sequences from across the Onchocercidae family. The *D. asiatica* clade is highlighted in light blue. Phylogenetic inference was made using a Bayesian and NJ distance method for a 465 bp segment of the filarioid mitochondrial *cox1* gene. Posterior probability values and bootstrap support (where available) for tree branches are indicated, with *Brugia malayi* used as an outgroup. Asterisked species names refer to sequences that were originally annotated as *Dirofilaria* sp. 'hongkongensis'; however, this taxon has now been formally described as *D. asiatica* (16).

A 465 bp stretch of the filarioid *cox1* gene from the *D. asiatica* sequences from four Cambodian dogs was used to construct a phylogenetic tree to assess the diversity of this taxon at the *cox1* locus and explore their relationship to other *Dirofilaria* species (Fig. 2). The *D. asiatica* sequences from Cambodia cluster with good support (posterior probability of 0.97 and bootstrap support of 99%) to numerous other *D. asiatica* sequences from Bhutan, Hong Kong, India, and Sri Lanka. Additionally, our sequences clustered separately from the closely related filarioid *D. repens* with a good posterior probability of 0.97 (bootstrap support was 65%). Some *D. asiatica* intraspecific diversity was also observed across our Cambodian infections with sequences from two canines forming a small sub-clade within the broader *D. asiatica* clade.

### Sequence type network

**Fig 3:**
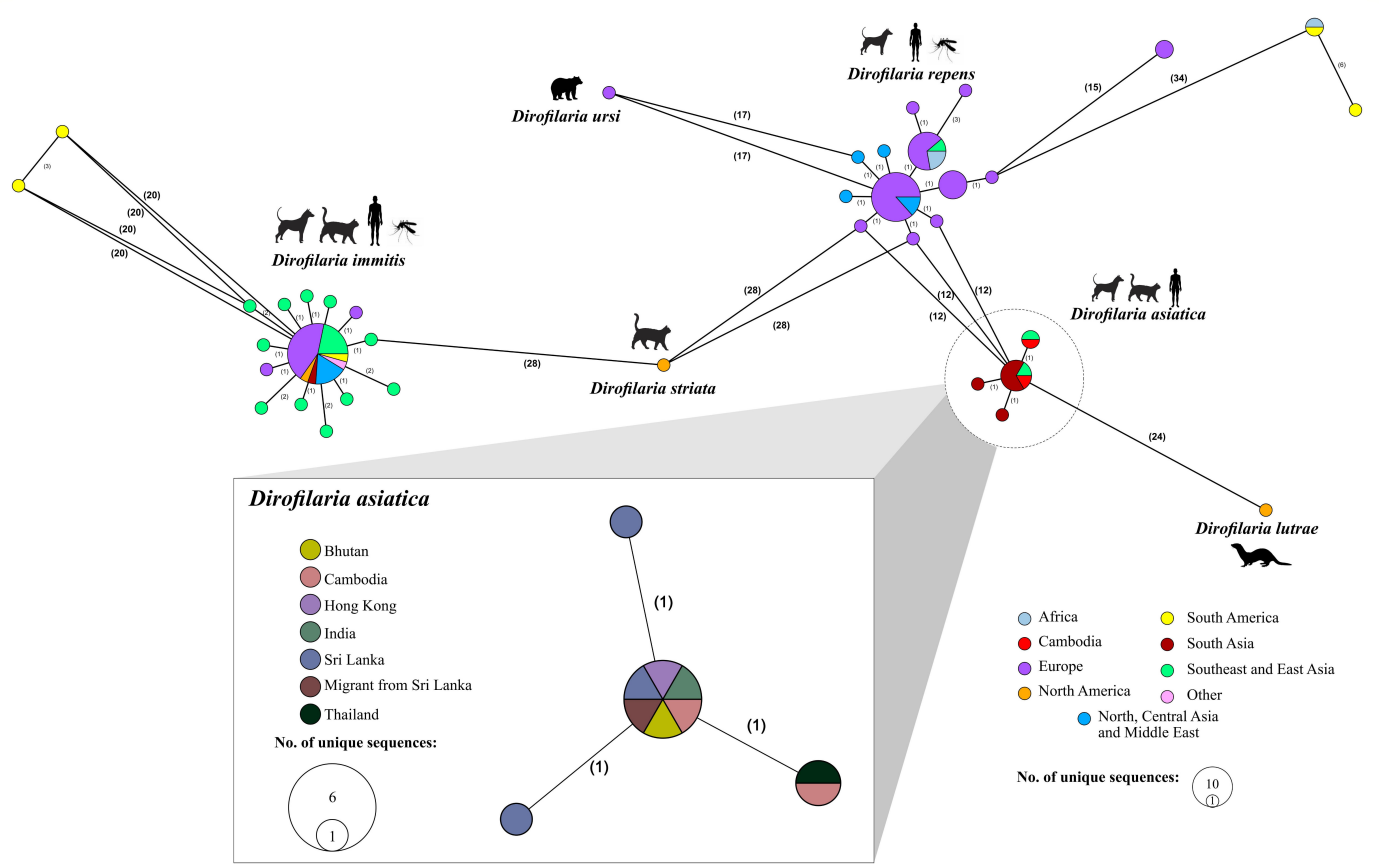
Minimum spanning network illustrating the unique sequence types (ST) using a 333 bp region of the mitochondrial *cox1* gene of *Dirofilaria* (main image) and *Dirofilaria asiatica* (insert) from the GenBank database. The number of nucleotide differences between each ST is indicated in parentheses, and the host species from which the *Dirofilaria* sequences were detected is indicated by silhouettes.

A total of 418 published and verified sequences were downloaded from NCBI’s GenBank database. From these, a total of 94 unique sequences based on location and haplotype were identified and extracted due to many sequences being replicates found across multiple studies. These sequences were aligned using a 333 bp region of the mitochondrial *cox1* gene found across all 94 extracted sequences. For *D. asiatica*, 10 unique sequences in terms of location and haplotype were identified. The MSN network of each *Dirofilaria cox1* ST is shown in Fig. 3.

## DISCUSSION

This study provides a comprehensive epidemiological exploration of filarioid parasites from dogs in Cambodia employing an advanced metabarcoding assay. The prevalence of filarioids in Cambodian dogs was found to be low at just 1% across the 504 individuals tested, with the majority caused by the zoonotic species *D. asiatica*. Previous research has identified increasing age and male sex as being significant predictors for canine dirofilariosis by species such as *D. asiatica* and others (3, 35). Within our study, two *D. asiatica* positive canines were below one year of age, potentially representing early infections, whilst 75% of the *D. asiatica* positive dogs were male. Importantly, the detection of *D. asiatica* in Cambodia represents a range extension for this parasite, which has only been detected in one other Southeast Asian nation before; Thailand, with the identification of a *D. asiatica*-like filarioid from a human patient (36).

Within the present study, we found that filarioid detection in Cambodia was clustered, with all *D. asiatica* found in the central eastern province of Tbong Khmum. European studies have found that *Dirofilaria* distribution can be highly localized within a country, with foci of high filarioid prevalence likely generated by environmental conditions conducive to mosquito vector reproduction and survival (11, 37). Such findings are mirrored by data from Cambodia, whereby the molecular identification of high levels of canine *D. immitis* infections (15.8%; *n* = 101) in ~2016 was not reflected in the data accrued through this study. This is likely due to the survey by Inpankaew (25) being conducted in the northeastern province of Preah Vihear, an area with different local climatic conditions than those presently explored. Importantly, the study by Inpankaew (25) does not present any sequencing data for the *D. immitis* detections identified, and therefore, it must be assumed that the detections in this study were inferred through a PCR band on a gel. It is notable that further studies completed since that by Inpankaew (25) have not been able to find evidence of *D. immitis* infections in Cambodian canines. For example, Huggins (22) tested 467 canine blood samples from the same study sites as those investigated here and found that no canines were antigen positive for *D. immitis* through serological testing conducted using the IDEXX SNAP 4D× Plus Test. Moreover, epidemiological discrepancies between the different studies may be further explained due to the filarioid nematode-targeting PCR used by Inpankaew (25, 38) having been developed before the discovery of *D. asiatica* as a cryptic and distinct species from *D. repens.* Therefore, whether this PCR can detect this filarioid species remains unknown, potentially explaining the lack of *D. asiatica* detection by Inpankaew (25).

The zoonotic nature of *D. asiatica* means that its detection within Cambodian canines is of high importance, given that this species may act as a silent and untreated reservoir of this human pathogen (3, 8, 14, 39). While canines infected with *D. asiatica* show little-to-no pathology (3), in humans, this filarioid can cause painful subcutaneous and subconjunctival nodules, ocular inflammation and related complications, as well as serious anaphylactic reactions after surgical removal of adult worms, as occurred to one infected patient in Hong Kong (5–8, 21, 40, 41). While there have been no reports of human *D. asiatica* cases in Cambodia to date, there is limited disease reporting within the country, while the symptoms generated by this parasite can easily be mistaken for other diseases or be ignored (5, 6, 42, 43). Notably, from 2005 onwards, the use of mass drug administration (MDA) programs employing albendazole and diethylcarbamazine was used to attempt to control lymphatic filariasis within Cambodia (44). Due to the success of this lymphatic filariasis control program, the World Health Organization declared Cambodia as having eliminated this disease as a public health problem in 2016, resulting in the cessation of MDA (44). Given that the canine reservoir of *D. asiatica* had not previously been detected nor addressed, the current lack of MDA leaves human populations in Cambodia particularly vulnerable to this and other zoonotic parasites.

Phylogenetic placement (Fig. 1) further confirmed the identity of four of the five filarioids detected from Cambodian canines as being *D. asiatica*. Such phylogenetic analyzes also highlighted the presence of low intraspecific genetic variation within this species in Cambodia. This intraspecific diversity is lower than that observed for *D. repens,* potentially due to *D. repens* being more adequately investigated with a greater representation of *cox1* gene sequences in GenBank. Previous studies have directly explored the genetic diversity of *D. repens,* identifying 18 haplotypes across Europe based on analysis of mitochondrial DNA sequences, with as many as 15 found in a single endemic country (45, 46). Given that *D. asiatica* is a close relative of *D. repens,* it may also exhibit such genetic diversity; however, this may not have been detected yet due to the paucity of research on this species. Most *D. asiatica* sequence types (ST) belonged to the same haplotype, with some exceptions, including a unique haplotype, comprising sequences from *Dirofilaria* in dogs from Cambodia (this study) and a case of periorbital dirofilariasis in a human from Thailand (GenBank accession: PP442028.1). Importantly, sequencing data and phylogenetic analyzes have also been used to detect other, as yet uncharacterized, *Dirofilaria* species from companion animals in Southeast Asia (16, 36). For example, *D. repens*-like nematodes have been identified, including *Dirofilaria* sp. 'Thailand II' from cats, amongst others, underscoring our still inchoate understanding of filarioid diversity within the region (16, 36, 47).

The identification of *A. reconditum* in one dog is also a novel report for Cambodia. This parasite causes subcutaneous filariosis in canines, with adult worms residing in subcutaneous tissues of the back, hind legs, and more rarely, the trunk (4, 48). Transmitted by fleas, *A. reconditum* is not typically thought to be clinically significant, with only rare reports of infections causing disease in dogs due to aberrant worm migration and localization (4, 49).

A metabarcoding approach was chosen to characterize canine filarioid nematode diversity in Cambodia, as such methods have demonstrated an improved ability to detect coinfections as well as rare, cryptic, or novel pathogens when compared to other diagnostic methods (22, 26, 50, 51). Importantly, in countries with scant prior data on pathogen epidemiology, traditional molecular methods, such as conventional and quantitative PCR, are sub-optimal as these methods can only detect one-to-a-few organisms per assay, with accurate selection of these requiring *a priori* knowledge of expected pathogens (22, 50, 52). This may, in part, be responsible for the limited epidemiological data that have to date been obtained for *D. asiatica* (16). In contrast, recent employment of novel metabarcoding methods has shown they can comprehensively characterize all species of pathogen, including those that are cryptic, from a given pathogen group (26, 53–55). Some of these methods have already been used in poorly investigated countries, such as Bhutan, Cambodia, and Mongolia, to unearth diverse and unexpected VBP species that would otherwise have been missed using traditional molecular techniques (22, 56, 57).

Within this study, the low number of filarioid-positive dogs meant that the large majority of deep-sequencing reads accrued were from the positive controls (95.7%). This is expected due to these controls comprising a specific and concentrated synthetic DNA sequence that is easily amplified by the metabarcoding assay’s primers to ensure the assay has worked and that a read cut-off threshold can be obtained (26) (26). Nanopore sequencing of the pre-screened positive samples found that those that were filarioid negative had sequences from off-target organisms such as canine genomic DNA.

To prevent canine infection by filarioid species, chemoprophylaxis by a microfilaricidal agent, e.g., moxidectin or milbemycin oxime, in conjunction with a chemical repellent effective against arthropods, e.g., imidacloprid and/or permethrin, is recommended (58–61). Such combinations have shown a high efficacy at protecting dogs from filarioid species, including *D. immitis* and *D. repens* (58, 59), and could likely be effective at protecting against *D. asiatica* as well. The use of topical chemopreventive products for dogs in tropical countries like Cambodia is also highly recommended due to their concomitant benefit in protecting against tick-borne pathogens, such as *Anaplasma platys*, *Babesia* spp., and *Ehrlichia canis* (62).

### Conclusions

This study provides the first comprehensive epidemiological data on filarioids infecting stray and semi-domesticated dogs from across Cambodia using a highly sensitive metabarcoding approach. Our data suggests a focal distribution of canine dirofilariosis, highlighting the need for future studies to define its range of distribution and explore environmental factors that may lead to local hotspots for different *Dirofilaria* species across Asia. This research also underscores the potential risk of *D. asiatica* infection to people in Cambodia and accentuates the need for increased awareness of this zoonotic and cryptic parasite by clinicians in the region, potentially in conjunction with targeted surveys to identify if this pathogen is emerging in local populations.

## Data Availability

The next-generation sequencing data that support the findings of this study are openly available in NCBI’s BioProject Database at accession number PRJNA1102149. Additionally, COI consensus sequences from canines found positive for *Dirofilaria asiatica* can be found in the NCBI nucleotide database under accession numbers PP843571, PP843572, PP843573, and PP843574.
